# Co-Delivery of Dihydroartemisinin and Indocyanine Green by Metal-Organic Framework-Based Vehicles for Combination Treatment of Hepatic Carcinoma

**DOI:** 10.3390/pharmaceutics14102047

**Published:** 2022-09-26

**Authors:** Yang Chen, Bin Wang, Wenping Chen, Tao Wang, Min Li, Zucheng Shen, Fang Wang, Jing Jia, Fenglan Li, Xiangyu Huang, Junyang Zhuang, Ning Li

**Affiliations:** 1 Department of Hepatobiliary Surgery, Fuzhou Second Hospital, Fuzhou 350007, China; 2 The Third Clinical Medical College, Fujian Medical University, Fuzhou 350007, China; 3 Fujian Key Laboratory of Drug Target Discovery and Structural and Functional Research, School of Pharmacy, Fujian Medical University, Fuzhou 350122, China; 4 Department of Oral and Maxillofacical Surgery, School and Hospital of Stomatology, Fujian Medical University, Fuzhou 350002, China

**Keywords:** ZIF-8, nanoparticles, dihydroartemisinin, phototherapy, combination therapy

## Abstract

Dihydroartemisinin (DHA), a widely used antimalarial agent, has clinical potential for the treatment of hepatic carcinoma. Although chemotherapy is indispensable for tumor therapy, it is generally limited by poor solubility, low efficiency, rapid clearance, and side effects. As an emerging treatment method, photothermal therapy (PTT) has many outstanding properties, but suffers from poor photostability of photosensitizer and incomplete ablation. Multimodal therapies could combine the advantages of different therapy methods to improve antitumor efficiency. Hence, we designed a nano-delivery system (ICG&DHA@ZIF-8) using zeolitic imidazolate framework-8 (ZIF-8) with a high porous rate and pH sensitivity property, to co-load DHA and indocyanine green (ICG). Dynamic light scattering and transmission electron microscopy were used to characterize the prepared nanoparticles. The photothermal conversion and drug release performances of ICG&DHA@ZIF-8 were investigated. In vitro antitumor efficacy and cellular uptake were studied. The mechanism of the combination treatment was studied by reactive oxygen species level detection and western blot assays. In vivo antitumor assays were then studied with the guidance of ex vivo imaging. The results showed that the ICG&DHA@ZIF-8 based combination therapy could efficiently kill hepatic carcinoma cells and suppress tumor growth. This research provides a potential nanodrug for the treatment of hepatic carcinoma.

## 1. Introduction

Dihydroartemisinin (DHA), a derivative of artemisinin, has widely been studied and used in malaria therapy [1]. DHA is recommended as the first-line antimalarial agent by the World Health Organization, but the expanding applications of DHA—such as an antitumor treatment—have attracted the attention of researchers [2]. As a small molecule drug, DHA has rapid blood clearance leading to a short blood half-life, which requires a high dosage or repeated administration for clinical application. Its nonspecific distribution may further lead to side effects [3]. Poor water solubility and low tumor accumulation are also limitations for the use of DHA in antitumor therapy. Therefore, various drug carriers, especially nanoscale vehicles, are being rapidly developed to overcome these limitations [4,5].

As one of the common traditional anticancer therapies, chemotherapy is indispensable for tumor therapy. However, patients usually suffer from serious side effects and unsatisfactory treatment efficiency [6]. These frustrating outcomes make it necessary to motivate the development of emerging therapies. Near-infrared (NIR) agents have recently been developed and employed for noninvasive photothermal therapy (PTT) [7,8]. PTT is a promising cancer treatment method that converts light into hyperthermia [9]. Specifically, photosensitizers can harvest the energy from a laser when they are exposed to laser irradiation, convert the light energy into heat, and then trigger the death of tumor cells accompanied by cellular overheating. PTT has many advantages, such as noninvasiveness, precise treatment, safety, and a high efficiency [10,11]. As an NIR agent clinically approved by the US Food and Drug Administration (FDA), indocyanine green (ICG) is considered safe, and offers an excellent in vivo biodegradability and light-specific toxicity in tumors [12,13]. However, ICG suffers from a poor photostability, short half-life, and nonspecific distribution [14]. These disadvantages make it necessary to develop suitable nanocarriers to improve the therapeutic efficiency of ICG.

The incomplete ablation of tumors caused by photothermal treatment alone limits photothermal therapeutic efficiency. As an emerging therapy approach, combination therapy with different drugs or treatment patterns could improve the therapeutic efficiency synergistically [15,16]. Combination therapy has been regarded as a hopeful strategy for its outstanding advantages. Chemotherapy and PTT are two different models of treatment. Research has shown that different models of treatment might provide synergistic effects [17]. Therefore, increasing attention has been focused on developing versatile vehicles to satisfy the needs of multimodal therapies [18]. 

In the last several decades, metal organic frameworks (MOFs) have emerged rapidly [19,20,21]. In particular, the field of MOFs as nanocarriers has developed rapidly, which has led to their increasing research in drug delivery areas [22,23]. As a classic porous MOF material, zeolitic imidazolate framework-8 (ZIF-8) is popular for its structures constructed from tetrahedral metal ions with imidazolate. ZIF-8 possesses unique advantages such as a high porous rate, high thermal and chemical stability, biomimetic mineralization and pH sensitivity [24,25,26]. These properties make ZIF-8 an attractive candidate for the encapsulation or adsorption of diverse bioactive substances (e.g., proteins, chemotherapeutic agents, and photosensitizers) under mild conditions, which is also termed biomimetic mineralization [24,25,26,27,28]. For example, Li et al. used ZIF-8 loaded with DHA for antitumor therapy [24]. Wang and colleagues encapsulated ICG in ZIF-8 for tumor fluorescence imaging and photothermal therapy [25]. Due to the instability of ZIF-8 under a weak acid environment, ZIF-8 possesses the pH-responsive and biodegradable properties for drug delivery and tumor therapy [28]. 

**Scheme 1:**
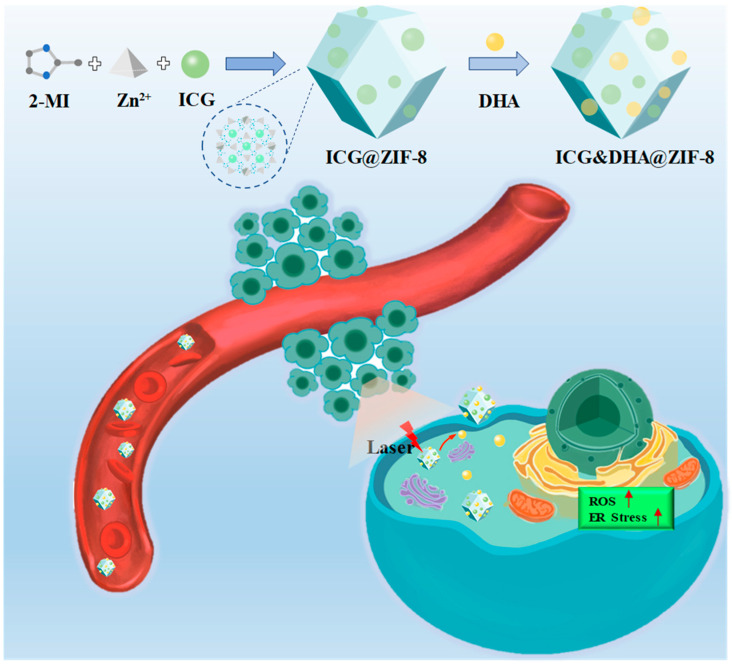
The schematic illustration of ICG&DHA@ZIF-8 nanoparticles mediated co-delivery of antitumor agent DHA and photosensitizer ICG. 2-MI: 2-nitroimidazole, ICG: indocyanine green. Upward arrow (↑) represents up-regulation of ROS level or ER stress.

In this work, we developed ZIF-8 nanoparticles as versatile vehicles to allow simultaneous encapsulation of DHA and ICG for the treatment of hepatic carcinoma. As shown in Scheme 1, the photosensitizer ICG was encapsulated by ZIF-8 through a one-pot biomineralized one-pot synthesis method and the agent DHA was subsequently absorbed under mild conditions, resulting in the formation of a nano-delivery system, ICG&DHA@ZIF-8. The properties of ICG&DHA@ZIF-8 such as morphology, size distribution and long-term stability were evaluated via dynamic light scattering (DLS) and transmission electron microscopy (TEM). Photothermal conversion tests and drug release assays were carried out to further study its physicochemical and pharmaceutical properties. In vitro assays were conducted to study the antitumor efficiency and the underlying mechanism. Under the guidance of ex vivo imaging, in vivo antitumor efficacy was studied. Co-delivery of DHA and ICG using ZIF-8 might provide a potential strategy for the treatment of hepatic carcinoma.

## 2. Materials and Methods

### 2.1. Materials

Dihydroartemisinin (DHA) and Nile Red were bought from Aladdin (Shanghai, China). 2-nitroimidazole (2-MI) and indocyanine green (ICG) were purchased from J&K Scientific (Beijing, China). Zinc nitrate and bovine serum albumin (BSA) were purchased from Sigma–Aldrich (Merck Life Science, Shanghai, China). 3-(4,5-dimethylthiazol-2-yl)-2,5-diphenyltetrazolium bromide (MTT), 4, 6-diamidino-2-phenylindole (DAPI), and 2′,7′-dichlorofluorescin diacetate (DCFH-DA) were bought from Beyotime Biotechnology (Shanghai, China). DMEM medium and fetal bovine serum (FBS) were bought from Shanghai Titan Scientific Co., Ltd., (Shanghai, China). CAAT/enhancer binding protein (C/EBP) homologous protein (CHOP), Bcl-2, and β-actin antibodies were bought from Cell Signaling Technology, Inc., (CST, Boston, MA, USA). 

Cells were kindly provided by Pricella Life Science&Technology Co., Ltd., (Wuhan, China).

### 2.2. Preparation and Characterization of ICG&DHA@ZIF-8 

ICG (1.3 mg, 1.7 μmol) and BSA (3 mg) were dissolved in imidazole solution. Then, under high-speed stirring, zinc nitrate solution (0.15 g/mL) was introduced dropwise (10 μL/min, 140 μL) into the mixture. The reaction was finished after 20 min, and the supernatant was collected after centrifugation (8000 rpm, 15 min). The precipitate was dispersed in ultrapure water. Afterward, ICG@ZIF-8 was harvested after centrifugation and washed with ultrapure water three times. 

DHA (2.6 mg, 9.1 μmol) was dissolved in a methanol solvent and sonicated for 30 min. Then, DHA was added into the ICG@ZIF-8 solution and kept at 50 °C overnight. After centrifugation (13000 rpm, 15 min, 4 °C) and washing with ultrapure water thrice, ICG&DHA@ZIF-8 was obtained and stored at 4 °C. Meanwhile, the crystal phases of the as-prepared ZIF-8 and ICG&DHA@ZIF-8 were investigated by X-ray diffraction (XRD) using a Cu target radiation resource after vacuum drying. N_2_ adsorption was tested at 25 °C using an ASAP 2460 analyzer (Micromeritics, Norcross, GA, USA) after pretreatment at 100 °C for 12 h in vacuum.

All supernatants during preparation were collected to detect the content of free drugs distributed among the supernatants. First, a part of the supernatants was dissolved in DMSO. Then, the absorption value was measured at 784 nm by UV spectrophotometry, and the concentration of free ICG in the supernatant was obtained. Then, the content of ICG loaded in ICG&DHA@ZIF-8 was obtained. Similarly, the supernatants were dispersed in an ethanol mixture solution (V_ethanol_:V_water_ = 1:4, containing 2% NaOH) and incubated in a water bath (60 °C) for 30 min. Then, the absorbance was measured at the wavelength of 238 nm by UV spectrophotometry, and the amount of DHA dispersed in the supernatant was acquired. Finally, the contents of the loaded drugs were calculated accordingly.

### 2.3. Size and Morphology

The DLS of ZIF-8, ICG@ZIF-8, and ICG&DHA@ZIF-8 was carried out using a Particle Analyzer (Anton Paar, Litesizer 500, Graz, Austria). The morphologies of the materials were observed by TEM (FEI, Tecnai G2, Hillsboro, OR, USA). To evaluate the long-term stability of the ICG&DHA@ZIF-8 solution, ICG&DHA@ZIF-8 was placed at room temperature for two weeks and the DLS data of the solution at set time points were monitored and acquired. 

### 2.4. Photothermal Conversion Effect of ICG&DHA@ZIF-8

The ICG&DHA@ZIF-8 (120 μg/mL of ICG) was dissolved in water and irradiated with a NIR laser (808 nm, Changchun Laser Optoelectronics Technology Co., Ltd., Changchun, China) for 5 min. Then, after natural cooling for 5 min to room temperature, the ICG&DHA@ZIF-8 solution was irradiated with the NIR laser (808 nm) for another 5 min. We repeated the irradiation and natural cooling process for three times. During the overall process, the temperature of the samples was monitored and recorded in real time by a pocket-portable thermal imaging camera (FLIR C3, Wilsonville, OR, USA). The ICG solution of the same equivalent concentration (120 μg/mL of ICG) was selected as a contrast.

### 2.5. Drug Release Assays

The drug release behavior was examined in buffers with different pHs and the contents of the released drug were detected through UV spectrophotometry. Specifically, ICG&DHA@ZIF-8 was dissolved in buffers (pH 5.4 or 7.4) and added into dialysis tubes (Molecular Weight Cut-off, MWCO = 3500). The tubes were immersed in vials containing the corresponding buffers and put in a shanker at 37 °C. Then, 450 μL of the buffer was taken out and detected using UV spectrophotometry at preset time points (1 h, 2 h, 3 h, 4 h, 5 h, 6 h, 8 h, 10 h, 12 h, 16 h, 24 h, and 36 h). The same volumes of fresh buffer were added into the vials. The released drug contents were calculated and the drug release curves were plotted. All the experiments were repeated three times.

### 2.6. In Vitro Antitumor Efficacy

A HepG2 cell line was chosen for the in vitro assays. HepG2 cells were cultured in DMEM medium (supplemented with 10% fetal bovine serum and 1% penicillin–streptomycin). The cells were first seeded in 96 wells and cultured for 24 h. After removing the media and washing with phosphate buffer solution (PBS), fresh medium containing different materials of a specific concentration was introduced. For ICG-based PTT, after incubation with ICG, ICG@ZIF-8, and ICG&DHA@ZIF-8 for 4 h, HepG2 cells were exposed to NIR (808 nm, 0.785 W/cm^2^) for 5 min and sequentially cultured. After incubation for another 44 h, the media were removed and the cells were washed with PBS twice. Then, the cell viability was evaluated through MTT assay.

### 2.7. Cellular Uptake Assays

HepG2 cells (5000 cells/well) were seeded in confocal dishes. After 36 h, Nile Red &DHA@ZIF-8 (concentration of Nile Red 80 ng/mL) was introduced. After co-incubation with Nile Red&DHA@ZIF-8 for a specific amount of time (1 h, 2 h, 4 h, 8 h), the culture media were discarded and the cells were rinsed with PBS twice. Then, the cells were fixed with paraformaldehyde for 15 min and washed with PBS thrice. Afterward, the cells were stained with DAPI for 5 min and washed with PBS thrice. The cells in PBS were observed on CLSM (Leica, SP5, Wetzlar, Germany).

HepG2 cells (1 × 10^5^) were seeded in 12 wells and incubated for 24 h. Then, they were cultured in a fresh medium containing Nile Red&DHA@ZIF-8 (concentration of Nile Red 80 ng/mL). After incubation for the set amount of time (1 h, 2 h, 4 h, 8 h), the cells were washed with PBS three times. Then, they were harvested and centrifuged (1000 rpm, 5 min). Finally, the cells dispersed in PBS were detected via flow cytometry (FCM, BD, Piscataway, NJ, USA).

For its suitable fluorescence spectrum, Nile Red was introduced to replace ICG for fluorescence detection on CLSM and FCM.

### 2.8. Cellular ROS Level Assays

HepG2 cells (10,000 cells/well) were seeded in confocal cell dishes. After 36 h, they were treated with free DHA, ICG@ZIF-8, and ICG&DHA@ZIF-8 (including 10 μg/mL DHA and 8 μg/mL ICG) for 4 h. Then, we removed the culture medium and washed the cells with PBS twice. Next, dichlorofluorescein diacetate (DCFH-DA) diluted in medium without phenol red solution was introduced to stain cells for 20 min. Afterward, the ICG@ZIF-8 group and the ICG&DHA@ZIF-8 group were irradiated with NIR (808 nm, 0.785 W/cm^2^) for 5 min. Subsequently, HepG2 cells were washed thrice with phenol–red–free medium. Finally, the cells were maintained in 200 μL PBS, and the expression of cellular ROS level was observed by CLSM.

### 2.9. Western Bolt Assays

HepG2 cells were seeded in 6 well plates. After 12 h, the cells were treated with free DHA, ICG@ZIF-8, and ICG&DHA@ZIF-8 for 24 h. The ICG@ZIF-8 group and the ICG&DHA@ZIF-8 group were irradiated with the NIR laser (808 nm, 0.785 W/cm^2^, 5 min) 4 h after drug administration. Afterwards, the cells were quickly collected and rinsed with PBS. For protein extraction, the cells were introduced to an appropriate amount of lysate and then transferred onto ice for 30 min. Subsequently, the solution was centrifuged (14,000 rpm, 4 °C, 15 min) to collect the supernatant. We used BCA protein assay kit (GlpBio, Montclair, CA, USA) to quantify the protein concentration. The 12% SDS-polyacrylamide gel electrophoresis (SDS-PAGE) was used to separate the protein. After that, the protein was transferred to the Poly (vinylidene fluoride) (PVDF) membranes, and the membranes were blocked with 5% skimmed milk for 1 h. Next, the membranes were incubated with the primary antibody, such as anti-CHOP and anti-Bcl-2, at 4 °C overnight. Then, we washed the PVDF membranes thrice with Tris buffered saline/Tween 20 (TBST) and put them on a shaker. They were incubated with the corresponding secondary antibodies for 1 h at room temperature. At the end of incubation, we used a gel imaging system (Bio-Rad, Hercules, CA, USA) to visualize these protein bands, and quantitative statistics on the bands’ intensity using ImageJ software.

### 2.10. Ex-Vivo Imaging

A tumor-bearing mouse model was established in line with reports [29]. After two weeks, the tumor-bearing mice were randomly divided into five groups, with three mice in each group. A quantity of 200 μL of ICG&DHA@ZIF-8 was injected by tail vein. Then, the mice were sacrificed at predetermined time points (0 h, 6 h, 12 h, 24 h, 36 h), and the tumors were collected for the imaging study using the IVIS Spectrum Imaging System (PerkinElmer, Waltham, MA, USA). The fluorescence was quantitatively analyzed using Living Image system software.

### 2.11. In Vivo Antitumor Efficiency

An HepG2 xenograft tumor model was established by subcutaneous implantation of 2 × 10^6^ tumor cells into the right abdomen of BALB/C nude mice (females, 6–8 weeks old). The tumor-bearing mice were randomly divided into four groups (three mice per group). The mice were injected every four days via the tail vein with PBS (control), free DHA, or ICG&DHA@ZIF-8 (dosage: 5 mg/kg of DHA, 4 mg/kg of ICG) with or without laser irradiation four times. The mice in the ICG&DHA@ZIF-8 with laser irradiation group were irradiated with the 808 nm laser (1.5 W/cm^2^, 5 min) 6 h after injection. Their tumor volumes and body weights were recorded every two days during a period of twenty-one days, while the values of the first day were set at 100%. At 21 days after treatment, the mice were sacrificed, and the tumors were resected and weighed to calculate the tumor growth inhibition (TGI) value according to the formula below: TGI (%) = (1 − mean tumor weigh of treated group/mean tumor weigh of control group) × 100% [30].

### 2.12. Statistical Analysis

All the data are present as Mean ± SD. The statistical analysis was calculated with student *t*-test by GraphPad Prism and *p* values < 0.05 were considered significant.

## 3. Results and Discussion

### 3.1. Characterization of ICG&DHA@ZIF-8

**Figure 1:**
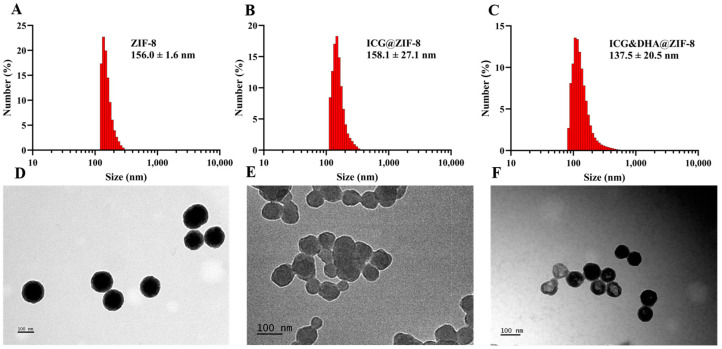
(**A**–**C**) Dynamic light-scattering results of ZIF-8, ICG@ZIF-8 and ICG&DHA@ZIF-8. (**D**–**F**) Representative transmission electron microscopy images of ZIF-8 (**D**), ICG@ZIF-8 (**E**), and ICG&DHA@ZIF-8 (**F**). Scale bars, 100 nm.

**Figure 2:**
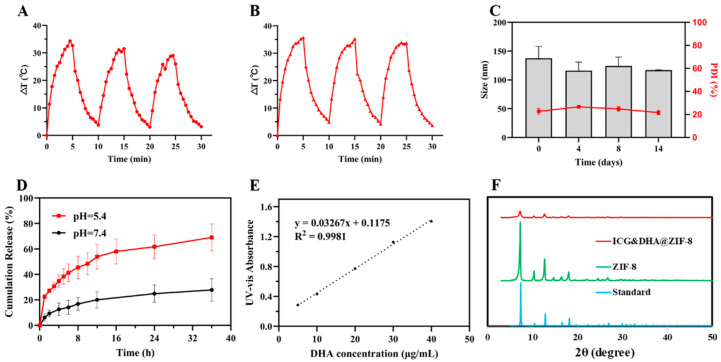
(**A**,**B**) The photothermal properties of free ICG and ICG&DHA@ZIF-8 (808 nm, 0.785 W/cm^2^). (**C**) The size variation and PDI of the ICG&DHA@ZIF-8 in water. (**D**) Cumulative DHA release from ICG&DHA@ZIF-8 in PBS at pH 7.4 and pH 5.4. (**E**) The standard curve of DHA content by UV-vis analysis. (**F**) PXRD patterns of ZIF and ICG&DHA@ZIF-8.

The size distribution and morphology of ZIF-8, ICG@ZIF-8 and ICG&DHA@ZIF-8 NPs were determined by DLS and TEM. As shown in the DLS measurements (Figure 1A–C), the average particle sizes of ZIF-8, ICG@ZIF-8, and ICG&DHA@ZIF-8 were 156.0 ± 1.6 nm, 158.1 ± 27.1 nm, and 137.5 ± 20.5 nm, respectively. The three nanoparticles showed narrow polydispersity indexes (PDI), which were 0.13, 0.08 and 0.23, respectively. In addition, the zeta potential of the three nanoparticles were −1.0 mV, −12.8 mV, and −19.4 mV, respectively (Figure S2). As shown in Figure 1D–F, the three ZIF-8 nanoparticles (NPs) exhibited a well-defined polyhedron shape and uniform particle sizes, consistent with the DLS measurements. Moreover, long-term stability tests showed that the diameters of ICG&DHA@ZIF-8 were 137.5 ± 20.5 nm, 116.0 ± 15.0 nm, 124.2 ± 15.5 nm, and 117.1 ± 0.5 nm at different time points (0, 4, 8, and 14 days, respectively) (Figure 2C). The results indicated that ICG&DHA@ZIF-8 could maintain its stability in water during two weeks at room temperature, with no obvious changes in particle size, because ZIF-8 formed by the coordination of Zn^2+^ and 2-methylimidazole (MIM) has excellent chemical and thermal stability [31]. These results might indicate the potentially good stability of the nanomedicines in blood circulation.

As for our prepared ZIF-8 and ICG&DHA@ZIF-8, their crystalline structures were evaluated by powder X-ray diffraction (PXRD). The PXRD pattern of our prepared ZIF-8 was the same as the standard [28]. After loading drug DHA and photosensitizer ICG, ICG&DHA@ZIF-8 showed an accordant pattern compared with the standard (Figure 2F). These findings indicated that loading ICG and DHA to ZIF-8 barely affected its crystalline structure. Porosity of the ZIF-8 and ICG&DHA@ZIF-8 was further analyzed by N_2_ physisorption. As shown in Figure S3, the surface area of ZIF-8 NPs was 469.3 m^2^/g. Its pore size distribution from 0 to 2 nm was calculated by the Horvath−Kawazoe (HK) method and the average pore size was about 0.93 nm (Figure S3). Interestingly, ICG&DHA@ZIF-8 maintained a similar average pore size to ZIF-8, while the surface area of ICG&DHA@ZIF-8 increased from 469.3 m^2^/g to 936.2 m^2^/g and the pore volume also increased from 0.17 cm^3^/g to 0.34 cm^3^/g. Meanwhile, the drug-loading capacities of DHA and ICG were detected using UV-Vis and calculated to be 16.7% and 10.9%, respectively. Additionally, the encapsulation efficiency of DHA and ICG were 25.2% and 62.5%, respectively.

### 3.2. Photothermal Properties of ICG&DHA@ZIF-8

ICG has been widely used in biological imaging, photothermal therapy (PTT), and photodynamic therapy (PDT), and its NIR light absorption characteristics could induce strong photothermal effects. However, its application is limited by poor stability, concentration quenching, photobleaching in an aqueous solution, and easy removal from the body [32]. To study the photothermal properties of ICG&DHA@ZIF-8 in an aqueous solution, the real-time changes of temperature were recorded at regular intervals by a thermal imager after NIR light irradiation. As shown in Figure 2B, the temperature of ICG&DHA@ZIF-8 increased to 63.7 °C rapidly within 5 min after irradiation with the 808 nm laser (0.785 W/cm^2^), indicating that ICG&DHA@ZIF-8 could quickly convert light into heat. Moreover, the temperature changes of ICG&DHA@ZIF-8 during three consecutive laser turn on/off cycles were 35.7 °C, 35.9 °C, and 34.3 °C, with no significant variation. However, the photothermal conversion capacity of free ICG gradually decreased during three cycles, with the temperature increment dropping from 34.3 °C to 29.0 °C (Figure 2A). Notably, ICG&DHA@ZIF-8 had higher photothermal conversion performance than free ICG. These results indicated that ICG&DHA@ZIF-8 had an excellent photostability and photothermal conversion efficiency.

### 3.3. pH Responsive DHA Release of ICG&DHA@ZIF-8

Controlled drug release at a tumor site is an effective strategy to optimize the efficacy of drug-targeted antitumor therapy by taking advantage of the characteristics of tumor microenvironments, such as weak acidity, high glutathione concentration, and overexpressed enzymes. The content of released drug was calculated using the standard curve (Figure 2E), and the cumulative release ratio of DHA from ICG&DHA@ZIF-8 at pH 7.4 or 5.4 PBS was obtained. As shown in Figure 2D, the DHA was released from the drug carrier rapidly, and almost 40% of DHA was found to release in 6 h. Finally, the cumulative release amount reached 69.1% after incubation for 36 h at pH 5.4 PBS. This phenomenon may be due to the rapid decomposition of the metal–ligand bonds of the ZIF-8 NPs in acidic conditions [28,33]. In contrast, little DHA was found to leak from the nanoparticles, and only 27.8% of DHA was released at the same time point (36 h) at pH 7.4 PBS. In addition, the release results of ICG also showed pH-responsive release characteristics (Figure S4). These results demonstrated the potential of ICG&DHA@ZIF-8 for long-term circulation stability with low side effects and tumor-specific pH-responsive drug release in vivo.

### 3.4. Cellular Uptake and Cytotoxicity of ICG&DHA@ZIF-8

**Figure 3:**
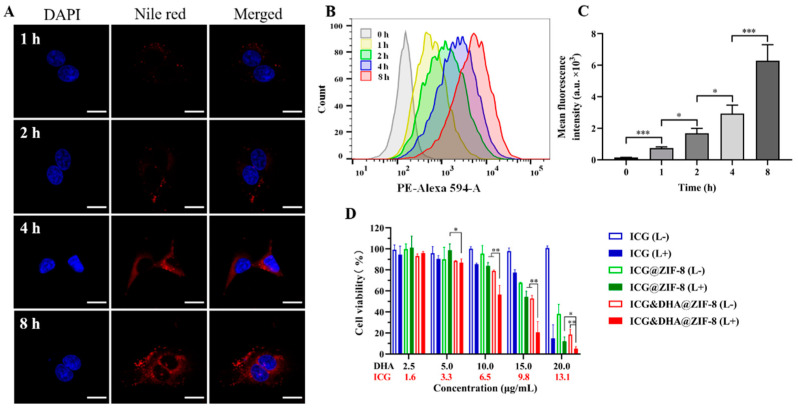
(**A**) Representative confocal microscopic images of cellular uptake after co-incubation with Nile Red&DHA@ZIF-8 for a specific amount of time (1 h, 2 h, 4 h, and 8 h). Scale bars, 20 μm. (**B**–**C**) Flow cytometry and its quantitative results of cellular uptake after co-incubation with Nile Red&DHA@ZIF-8 for a specific amount of time (1 h, 2 h, 4 h, and 8 h) (*n* = 3). (**D**) Relative cell viability of HepG2 cells after incubation with free ICG, ICG@ZIF-8, and ICG&DHA@ZIF-8 in the presence/absence of 808 nm light irradiation (“(L−)” means without laser irradiation, “(L+)” means with laser irradiation). * *p* < 0.05, ** *p* < 0.01, *** *p* < 0.001.

The cellular uptake and cytotoxicity of ICG&DHA@ZIF-8 were estimated in the human hepatocellular carcinoma (HCC) HepG2 cell line. To confirm the cell internalization by CLSM and FCM, Nile Red was embedded into the ZIF-8 NPs instead of ICG for the convenience of fluorescence detection. Nile Red&DHA@ZIF-8 showed a diameter of 169.7 nm with a zeta potential of −14.6 mV (Figures S1 and S2). CLSM images showed that bright red fluorescence in cells was observed after incubation for 2 h, and the fluorescence intensity increased over time (Figure 3A). Moreover, the flow cytometry analysis also showed that the cellular uptake of ZIF-8 NPs was evidently time-dependent (Figure 3B,C). These results indicated that ICG and DHA could be successfully and efficiently delivered into cells via ZIF-8 NPs, thereby exerting antitumor effects in cells.

The pH-responsive DHA release, excellent photostability and photothermal conversion efficiency, with the addition of efficient cell internalization, imply that ICG&DHA@ZIF-8 might have outstanding antitumor potential. The cytotoxicity of free DHA, free ICG, ZIF-8, ICG@ZIF-8, and ICG&DHA@ZIF-8 was evaluated after incubation with HepG2 cells for 48 h, while the MTT method was chosen to calculate relative cell viability. As shown in Figure S5, the half maximal inhibitory concentration (IC50) values were 7.3 μg/mL for free DHA and 12.3 μg/mL for ICG&DHA@ZIF-8 without light irradiation. Unlike free DHA, which is able to rapidly enter cancer cells through passive diffusion, the uptake of ZIF-8 NPs by cancer cells depends on endocytosis, which is a relatively slower process. To further evaluate the toxicity of ICG&DHA@ZIF-8 against HepG2 cells in the presence of a laser, the cells were incubated with ICG&DHA@ZIF-8 for 4 h, and then irradiated with the 808 nm laser (0.785 W/cm^2^, 5 min). Free ICG and ICG@ZIF-8 were applied as control. As shown in Figure 3D, free ICG exhibited no obvious toxicity without irradiation and needed to reach a certain concentration to kill cells under laser irradiation. In addition, the relative cell viability of ICG@ZIF-8 was lower than free ICG in the presence of a laser due to ZIF-8 NPs improved the photostability and photothermal conversion efficiency of ICG. As expected, at the same concentration of DHA and ICG, the relative cell viability of ICG&DHA@ZIF-8 with laser irradiation was significantly reduced compared to ICG&DHA@ZIF-8 without laser irradiation and ICG@ZIF-8 with laser irradiation. Notably, ZIF-8 NPs showed no significant toxicity even at the concentration of 80 μg/mL, indicating that ZIF-8 NPs has good biocompatibility (Figure S6). These results suggest that the combination of cytotoxicity of DHA and photo-cytotoxicity of ICG could significantly enhance the tumor-therapeutic effect of ICG&DHA@ZIF-8.

### 3.5. ROS Level Detection

**Figure 4:**
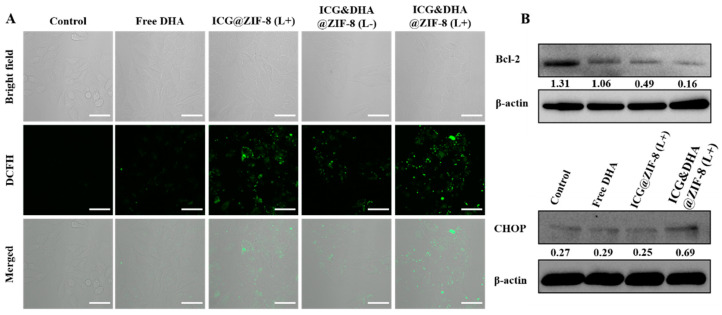
(**A**) Representative confocal microscopic images of intracellular ROS production after incubation with free DHA, ICG@ZIF-8, and ICG&DHA@ZIF-8 with or without laser irradiation (“ICG@ZIF-8 (L+)” means ICG@ZIF-8 with laser irradiation, “ICG&DHA@ZIF-8 (L−)” means ICG&DHA@ZIF-8 without laser irradiation, “ICG&DHA@ZIF-8 (L+)” means ICG&DHA@ZIF-8 with laser irradiation). Scale bars: 50 nm. (**B**) Western blot analysis of CHOP and Bcl-2 protein expression in HepG2 cells treated with free DHA, ICG@ZIF-8, and ICG&DHA@ZIF-8 with laser irradiation.

Cellular ROS has a significant impact in tumor therapy. An increased ROS level might induce oxidative stress to cause damage to biological macromolecules such as nucleic acids, proteins, and cell membranes [34]. The fluorescence probe of dichlorodihydrofluorescein diacetate (DCFH-DA) has been widely applied for intracellular ROS detection. Hence, a DCFH-DA probe was applied to detect the variation of the intracellular ROS level in HepG2 cells after treatment with DHA, ICG@ZIF-8, and ICG&DHA@ZIF-8 with or without laser irradiation. As shown in Figure 4A and Figure S7, in the absence of laser irradiation, free DHA could slightly induce ROS production, whereas ICG&DHA@ZIF-8 could induce higher ROS levels than free DHA due to sustained release of the drug to stimulate cells. In addition, ICG@ZIF-8 with laser irradiation significantly enhanced ROS production compared with the control group. When exposed to the same laser irradiation, ICG&DHA@ZIF-8 further significantly increased the level of ROS with the combined help of DHA and ICG. These results suggest that DHA and PTT could both induce the intracellular ROS level increased.

In addition, an increased ROS level has the potential to generate anomalous elevation of unfolded proteins, thereby leading to endoplasmic reticulum (ER) stress [35].

### 3.6. Western Blot Assays

Perturbation of ER homeostasis affects protein folding and causes ER stress [36]. CAAT/enhancer-binding protein homologous protein (CHOP) is also known as growth arrest-and DNA damage-inducible gene 153 (GADD153). CHOP/GADD153 is one of the components involved in ER stress-mediated apoptosis, and the elevated expression of CHOP is considered a marker of ER stress [37]. Additionally, Bcl-2 has also been reported to be related to ER stress [38]. Western blot assays were carried out to study the expression of proteins such as CHOP and Bcl-2. As shown in Figure 4B, CHOP expression in the group of ICG&DHA@ZIF-8 with laser irradiation was significantly higher than that in the control group. The group treated with ICG&DHA@ZIF-8 with laser irradiation suppressed the expression level of anti-apoptotic Bcl-2. Overall, these results suggest that ICG&DHA@ZIF-8 with laser irradiation induces ER stress. Therefore, for ICG&DHA@ZIF-8, the combination of DHA treatment and PTT enhances cytotoxicity by inducing the increase in ROS level and upregulating ER stress. These results suggest that ICG&DHA@ZIF-8 might be a good candidate system for cancer therapy.

### 3.7. Ex Vivo Tumor Imaging Study

**Figure 5:**
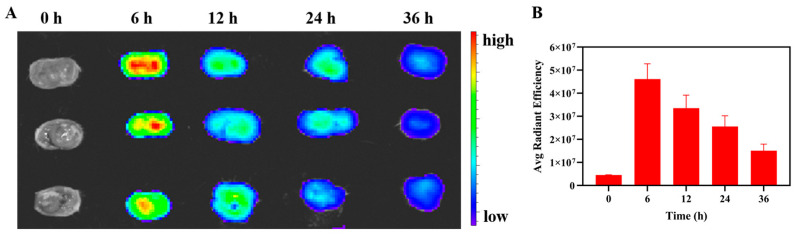
(**A**) Ex vivo fluorescence imaging of tumors at different time points after injection of ICG&DHA@ZIF-8 by tail vein. (**B**) Average radiant efficiency fluorescence values of tumors (*n* = 3).

To further evaluate the tumor accumulation of ICG&DHA@ZIF-8, ex vivo tumor imaging was studied. As shown in Figure 5A, the bright fluorescence signal of ICG in the tumor could be clearly observed at 6 h, and even the ICG fluorescence could be observed continuously for 36 h. In addition, the quantitative analysis results showed that the fluorescence intensity of ICG was the strongest at 6 h, and the fluorescence intensity at 36 h was still one third of that at 6 h (Figure 5A,B). These results indicated that ICG&DHA@ZIF-8 could quickly accumulate in tumor, and retain in tumor for an extended period. Moreover, these results might offer image guidance for in vivo antitumor therapy.

### 3.8. In Vivo Evaluation of the Antitumor Effect

**Figure 6:**
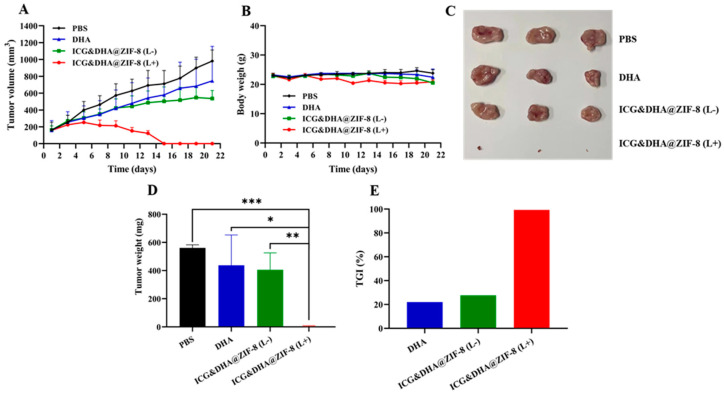
(**A**,**B**) Curves of tumor volume growth and body weight changes during the period of 21 days for all groups (“ICG&DHA@ZIF-8 (L−)” means ICG&DHA@ZIF-8 without laser irradiation, “ICG&DHA@ZIF-8 (L+)” means ICG&DHA@ZIF-8 with laser irradiation) (*n* = 3). (**C**,**D**) Photos and weight of the tumors after all of the treatments (*n* = 3). (**E**) TGI of the three treatment groups. * *p* < 0.05, ** *p* < 0.01, *** *p* < 0.001.

On the basis of the above in vitro antitumor and ex vivo imaging results, we further studied the in vivo therapeutic effects of HepG2 tumor-bearing mice treated with PBS, free DHA, and ICG&DHA@ZIF-8 with or without laser irradiation. As shown in Figure 6A, the tumor growth of the free DHA group was only slightly inhibited compared with the PBS group, and ICG&DHA@ZIF-8 without laser further delayed tumor growth compared with free DHA. At the equivalent dosage of DHA and ICG, tumor volume in the group of ICG&DHA@ZIF-8 with laser showed a decreasing trend after laser irradiation and these tumors were almost completely ablated after four treatments. The body weight of mice was recorded every two days during the period of 21 days and no significant changes were observed in any of the groups (Figure 6B). In addition, tumors were collected and weighed, and the tumor growth inhibition value (TGI) was calculated. As shown in Figure 6C–E, the mean tumor weight of the control group was 561.2 ± 21.8 mg. The mean tumor weight of free DHA and ICG&DHA@ZIF-8 were 437.5 ± 215.5 mg and 405.6 ± 119.8 mg, while their TGI values were 22.0% and 27.7%, respectively. Notably, the mean tumor weight of the ICG&DHA@ZIF-8 with laser irradiation group was 3.8 ± 4.5 mg, and the TGI reached 99.3%. The ICG&DHA@ZIF-8 with laser irradiation exhibited significant inhibition of tumor growth and therapeutic effects, which may be the result of the combination of DHA and PTT. All of these results indicated that ICG&DHA@ZIF-8 NPs could achieve long circulation in vivo, accumulate in tumors, be effectively internalized into tumor cells, and pH-responsively release the drug DHA and the photosensitizer ICG, and finally improve the therapeutic efficiency in combination with PTT and chemotherapy.

## 4. Conclusions

We successfully established a co-delivery system based on ZIF-8 for hepatic carcinoma treatment. ICG and DHA were loaded into ZIF-8 via a one-pot biomineralization method and adsorption strategy. The DLS and TEM results showed that the processed ICG&DHA@ZIF-8 nanoparticles had a uniform morphology, size, and long-term stability. The improved photostability and excellent photothermal conversion efficiency of ICG&DHA@ZIF-8 compared with free ICG were proven by photothermal conversion assays. A series of in vitro studies showed that ICG&DHA@ZIF-8 could be uptaken by tumor cells quickly and responsively release the loading drug DHA and the photosensitizer ICG under the intracellular acid environment. As DHA and PTT could increase the cellular ROS level and induce ER stress, an antitumor effect was directly achieved. Moreover, by coupling with the ICG-mediated PTT strategy, the antitumor effect was further enhanced for killing hepatic carcinoma cells. Attributed to good long-term stability, improved photostability, excellent photothermal conversion efficiency, good accumulation in tumor, rapid internalization, pH-responsive intracellular drug release, an increased ROS level, and induced ER stress, the ICG&DHA@ZIF-8 system on ZIF-8 showed excellent in vivo antitumor efficiency with the combination of chemotherapy and PTT. Inspiringly, tumors were almost ablated in the group treated with ICG&DHA@ZIF-8 with laser irradiation. This work offers a promising alternative strategy for the treatment of hepatic carcinoma.

## Data Availability

Not applicable.

## Supplementary Materials

The following supporting information can be downloaded at: https://www.mdpi.com/article/10.3390/pharmaceutics14102047/s1, Figure S1. Dynamic light scattering results of Nile Red&DHA@ZIF-8. Figure S2. Zeta potential of ZIF-8, ICG@ZIF-8, ICG&DHA@ZIF-8, and Nile Red&DHA@ZIF-8. Figure S3. N_2_ adsorption isotherms and Horvath−Kawazoe (HK) pore size distribution of ZIF-8 and ICG&DHA@ZIF-8. Figure S4. Cumulative ICG release from ICG&DHA@ZIF-8 in PBS at pH 7.4 and pH 5.4. Figure S5. Relative cell viability of HepG2 cells after incubation with free DHA and ICG&DHA@ZIF-8 (L−). Figure S6. Relative cell viability of HepG2 cells after incubation with ZIF-8. Figure S7. Fluorescent quantitative analysis of the ROS level.
