# Supplementary material for: Co-Delivery of Dihydroartemisinin and Indocyanine Green by Metal-Organic Framework-Based Vehicles for Combination Treatment of Hepatic Carcinoma
Source: Pharmaceutics. 2022 Sep 26;14(10):2047. doi: 10.3390/pharmaceutics14102047 (PMC9610498; doi:10.3390/pharmaceutics14102047)
Supplement: Supplementary file 1 [file pharmaceutics-14-02047-s001.zip › pharmaceutics-1868013-supplementary.pdf]

Supplementary Information

Co-delivery of dihydroartemisinin and indocyanine green by metal-organic framework-based vehicles for combination treatment of hepatic carcinoma

Yang Chen [1,,†], Bin Wang [3,†], Wenping Chen [3], Tao Wang [4], Min Li [3], Zucheng Shen [3], Fang Wang [3], Jing Jia [3], Fenglan Li [3], Xiangyu Huang [3], Junyang Zhuang [3,*] and Ning Li [3,*]

[1] Department of Hepatobiliary Surgery, Fuzhou Second Hospital, Fuzhou 350007, China.

[2] The Third Clinical Medical College, Fujian Medical University, Fuzhou 350007, China.

[3] Fujian Key Laboratory of Drug Target Discovery and Structural and Functional Research, School of Pharmacy, Fujian Medical University, Fuzhou 350122, China.

[4] Department of Oral and Maxillofacial Surgery, School and Hospital of Stomatology, Fujian Medical University, , Fuzhou 350002, China.

* Corresponding author. E-mail: Junyang.Zhuang@hotmail.com (J. Z.); fjgtlny@126.com or ningli@fjmu.edu.cn (N. L.)

† These authors contributed equally to this work.

Table of Contents

Figure S1. Dynamic light scattering results of Nile Red&DHA@ZIF-8.

Figure S2. Zeta potential of ZIF-8, ICG@ZIF-8, ICG&DHA@ZIF-8, and Nile Red&DHA@ZIF-8.

Figure S3. N_2 adsorption isotherms and Horvath−Kawazoe (HK) pore size distribution of ZIF-8 and ICG&DHA@ZIF-8.

Figure S4. Cumulative ICG release from ICG&DHA@ZIF-8 in PBS at pH 7.4 and pH 5.4.

Figure S5. Relative cell viability of HepG2 cells after incubation with free DHA and ICG&DHA@ZIF-8 (L-).

Figure S6. Relative cell viability of HepG2 cells after incubation with ZIF-8.

Figure S7. Fluorescent quantitative analysis of the ROS level.

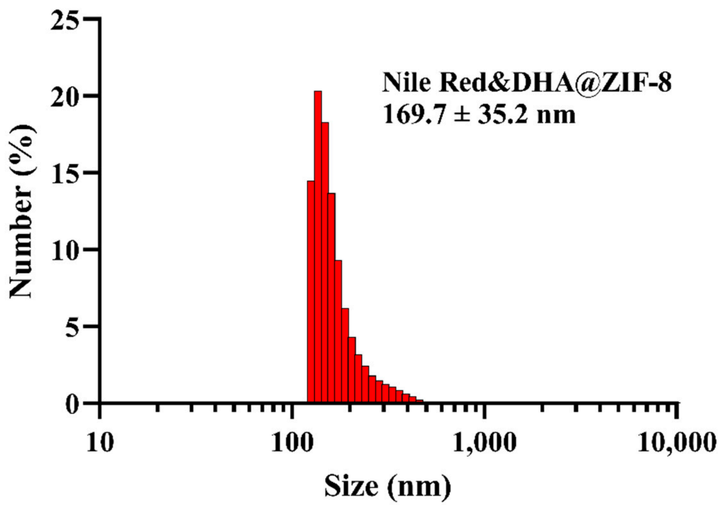

Figure S1. Dynamic light scattering results of Nile Red&DHA@ZIF-8.

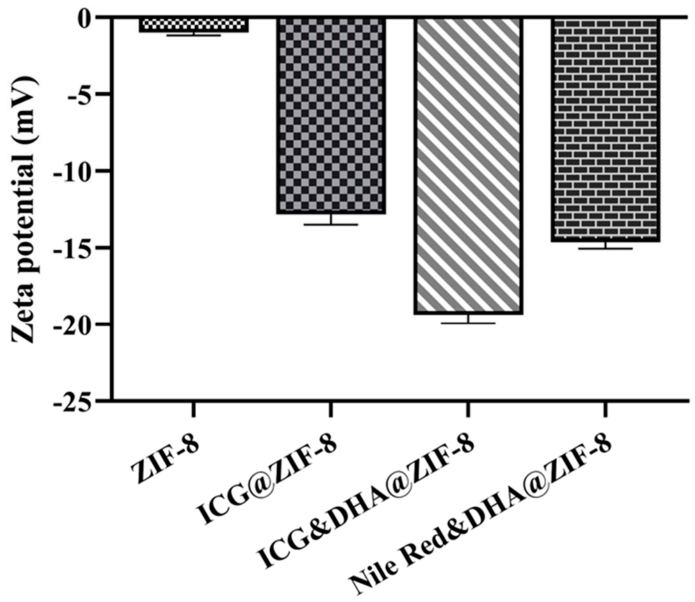

Figure S2. Zeta potential of ZIF-8, ICG@ZIF-8, ICG&DHA@ZIF-8, and Nile Red&DHA@ZIF-8.

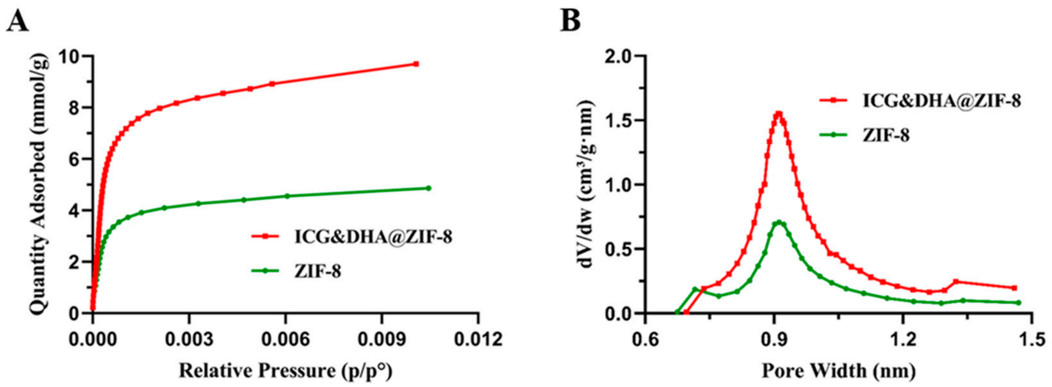

Figure S3. N_2 adsorption isotherms (A) and Horvath−Kawazoe (HK) pore size distribution (B) of ZIF-8 and ICG&DHA@ZIF-8.

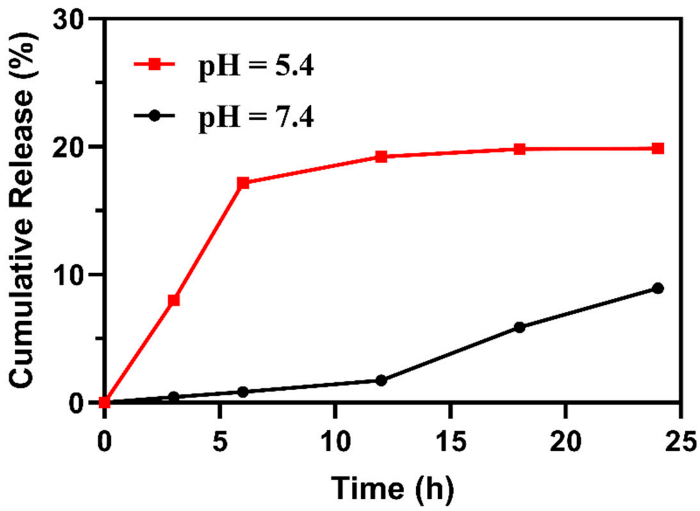

Figure S4. Cumulative ICG release from ICG&DHA@ZIF-8 in PBS at pH 7.4 and pH 5.4.

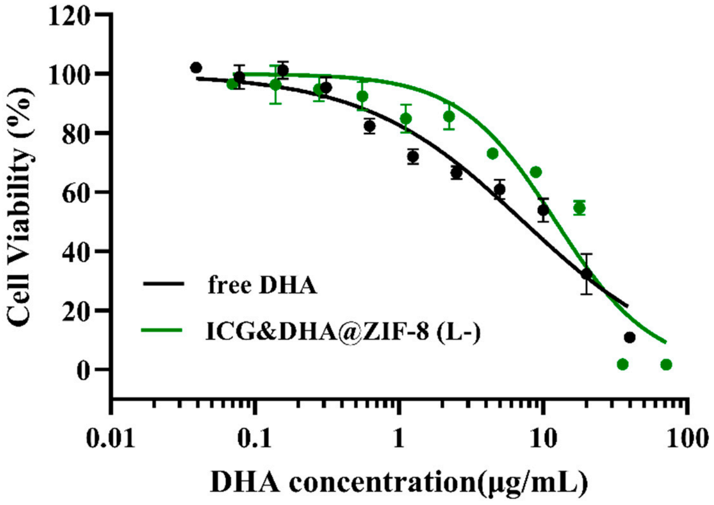

Figure S5. Relative cell viability of HepG2 cells after incubation with free DHA and ICG&DHA@ZIF-8 (L-) ("ICG&DHA@ZIF-8 (L-)" means ICG&DHA@ZIF-8 without laser irradiation).

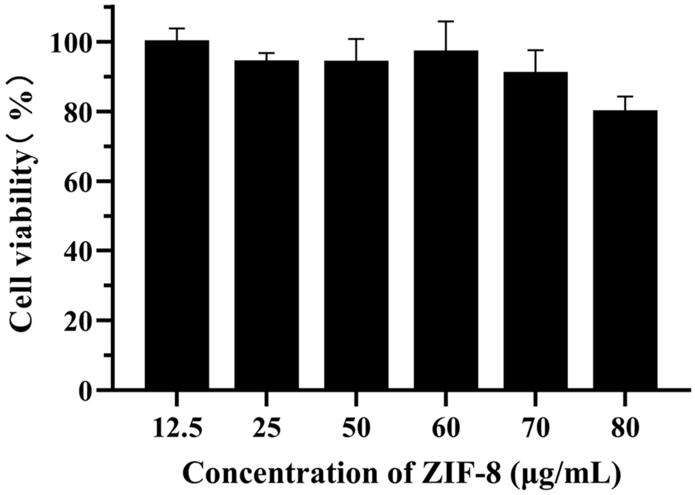

Figure S6. Relative cell viability of HepG2 cells after incubation with ZIF-8.

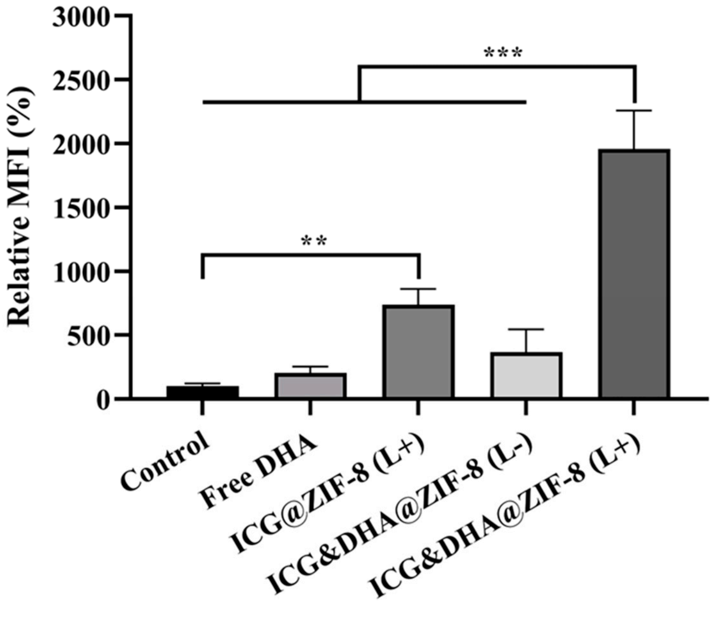

Figure S7. Fluorescent quantitative analysis of the ROS level ($n = 3$). *$*p <$ 0.05, $**p < 0.01$, $***p < 0.001$.*
